# Serotonin syndrome in the acute treatment landscape of migraine: the lasmiditan experience

**DOI:** 10.3389/fneur.2023.1291102

**Published:** 2023-10-27

**Authors:** Andrew Blumenfeld, Stewart J. Tepper, Rashna Khanna, Erin Doty, Maurice Vincent, Sheila I. Miller

**Affiliations:** ^1^The Neurology Center of Southern California, Carlsbad, CA, United States; ^2^Geisel School of Medicine at Dartmouth, Dartmouth-Hitchcock Medical Center, Lebanon, NH, United States; ^3^Eli Lilly and Company, Bracknell, United Kingdom; ^4^Eli Lilly and Company, Indianapolis, IN, United States

**Keywords:** migraine, lasmiditan, serotonin syndrome, 5-hydroxytryptamine-1F, drug interaction

## Abstract

**Background:**

Serotonin syndrome (SS) symptoms overlap with adverse events associated with lasmiditan, a 5-HT (serotonin)_1F_ receptor agonist for acute treatment of migraine. Because SS symptoms are heterogeneous, diagnosis can be challenging, and potential cases observed with lasmiditan treatment led to questions about SS pathophysiology. Here, we provide an overview of the potential risk of SS based on experience with lasmiditan.

**Methods:**

Results of eight phase 2 and phase 3 lasmiditan trials (*n* = 5,916) and a controlled intravenous trial of lasmiditan (*n* = 88) were analyzed for symptomatology consistent with SS. Post-marketing surveillance data from lasmiditan’s US launch date (January 2020) until data cut-off (April 2021) were also examined. Established Sternbach and Hunter diagnostic criteria were used for formal determination of SS.

**Results:**

Of 6,004 lasmiditan-treated clinical trial patients, 15 reported ≥1 treatment-emergent adverse event consistent with signs and symptom(s) of SS. After review, one case met Sternbach and Hunter criteria, two cases potentially met Sternbach criteria, and three cases reported as SS had limited/no information to determine if either criterion was met. During post-marketing surveillance (approximately 13,400 lasmiditan prescriptions), 17 cases with symptom complexes consistent with SS were reported; 3/17 cases had adequate case descriptions to apply predefined criteria. Of these, two met Sternbach and Hunter criteria, and one met Sternbach criteria.

**Conclusion:**

Awareness of clinical symptomatology and diagnostic criteria of SS can help clinicians with recognition of rare instances of SS that may occur with lasmiditan.

**Clinical trial registration:**

NCT03670810, NCT00384774, NCT00883051, NCT02565186.

## Introduction

*“There is no more difficult art to acquire than the art of observation, and for some men it is quite as difficult to record an observation in brief and plain language.”* — Osler, W (1).

Serotonin, or 5-hydroxytryptamine (5-HT), is a neurotransmitter classified as a biogenic monoamine similar to norepinephrine, dopamine, and histamine (2). Serotonin modulates neural activity and neurophysiological functions including sleep/wakefulness, affect and behavior, thermoregulation, pain, and cerebrospinal fluid synthesis. Outside of the central nervous system (CNS), serotonin is involved in several biological processes, including cardiovascular function, energy balance and food intake, bowel activity, bladder control, and platelet aggregation (3). Complexity of serotonergic signaling is attributed to 14 distinct receptors classified into 7 main families: 5-HT_1_ to 5-HT_7_ (Supplementary Table S1) (4). Dysfunction of the serotonergic system is involved in the pathophysiology of psychiatric and neurological disorders including migraine (5).

The role of serotoninergic signaling in the treatment of migraine was initially attributed to vasoconstriction. At the time, the serotonin receptors had not been fully characterized. The vasoconstriction hypothesis led to the development of sumatriptan, the first selective 5-HT_1_ agonist, with both 5-HT_1B_-mediated vasoconstrictive and 5-HT_1D_-mediated neuroactive effects (6). Sumatriptan’s mechanism of action revolutionized the treatment of migraine and served as a catalyst to elucidate the role of serotonin in migraine pathophysiology. After the discovery of this complex serotonergic signaling, which emphasized that vasoconstriction was not a required mechanism underlying treatment of a migraine attack, the evolution of triptans focused on 5-HT_1D_ and led to the development of targeted therapies.

### Serotonin syndrome, serotonin toxicity, and drugs associated with their development

Serotonin toxicity and syndrome are drug-induced events caused by an iatrogenic increase of intrasynaptic concentrations of serotonin in the central and peripheral nervous systems (7, 8). Serotonin toxicity can range from mild to severe based on the level of increased serotonin, with the intensity of the signs and symptoms reflecting the severity of the toxicity (9, 10). Mild cases of serotonin toxicity are underreported or often dismissed (11). On the other hand, the rarer and potentially life-threatening instances of serotonin toxicity are generally referred to as serotonin syndrome (SS) (5, 10).

The pathophysiology of SS is complex and involves various neurotransmitters. The predominant mechanism for SS involves either the increased availability of serotonin in the synapse or hyperstimulation of serotonin receptors (5-HT_1A_ > 5-HT_2A_) (5). Several drugs, alone or in combination, have been associated with the development of SS (9–12). Population-level data are available on the reported occurrence of SS with selective serotonin reuptake inhibitors (SSRIs), serotonin and norepinephrine reuptake inhibitors (SNRIs), and other serotonergic medications, used alone or in combination (5). Published data from the Toxic Exposure Surveillance System reported 7,350 cases of toxic side effects and 93 deaths on exposure to SSRIs (12). A report on the overdose of SSRIs indicated 14% of cases developed SS (13). Another study from the British National Health Service reported that the incidence of SS was 0.5–0.9 cases per 1,000 patient-months of treatment with SSRI monotherapy (14). Notably, 30% of patients on venlafaxine, an SNRI, develop serotonin toxicity (12). Drugs that are commonly associated with serotonin toxicity and syndrome, acting either alone or in combination with other drugs, are listed in Table 1 (5, 10). Triptans (5-HT_1B_ and 5-HT_1D_ agonists) and ditans (5-hydroxytryptamine-1F agonists), indicated for acute treatment of migraine, have been evaluated for SS, but the mechanism by which this might occur is unclear, as these selective agonists do not bind to 5-HT_1A_ or 5-HT_2A_ receptors (12). In 2006, the Food and Drug Administration (FDA) issued an alert against the use of combination of triptans with SSRIs or SNRIs based on their review of 27 cases. When these 27 cases and 2 subsequent cases were reviewed by Evans, the FDA-cited case reports revealed omission of critical clinical information, thus calling into question the accuracy of most assignments of the diagnosis of SS (15). Of the 29 cases obtained by the FDA, only 10 cases met the Sternbach criteria and none met the Hunter criteria (15). Even among those cases meeting the Sternbach criteria, some questions arose with several of the cases. The American Headache Society Position Paper (2010) on this issue concluded, “Data are inadequate or conflicting (15). Given current knowledge on the risks of combining triptans with SSRIs/SNRIs, increased risks of SS are unproven.” Subsequent published cases also failed on diagnostic criteria grounds to establish a causative relationship between triptans and SS (16, 17).

**Table 1:** Drug that have been shown to be associated with SS (5, 10).

Drug classes	Drugs listed but not limited to the following
Antidepressants/mood stabilizers	
Monoamine oxidase inhibitors (MAOIs)	Safinamide, safinamide, selegiline, rasagiline, phenelzine, tranylcypromine, isocarboxazid, moclobemide, linezolid, tedizolid, methylene blue, procarbazine
Selective serotonin reuptake inhibitors (SSRIs)	Citalopram, escitalopram, fluoxetine, fluvoxamine, paroxetine, sertraline
Serotonin-norepinephrine reuptake inhibitors (SNRIs)	Desvenlafaxine, duloxetine, milnacipran, venlafaxine
Dopamine-norepinephrine reuptake inhibitors (DNRIs)	Buspirone
Tricyclic antidepressants (TCAs)	Amitriptyline, amoxapine, clomipramine, desipramine, doxepin, imipramine, maprotiline, nortriptyline, protriptyline, trimipramine
Miscellaneous mechanisms	Lithium, St. John’s wort (*Hypericum perforatum*), trazadone
Antiemetics	Metoclopramide, ondansetron, granisetron
Antimigraine drugs	Triptans, carbamazepine, ergot alkaloids, valproic acid
Opioids	Levomethorphan, levorphanol, meperidine, methadone, tapentadol, tramadol
Second-generation antipsychotics	Aripiprazole, clozapine, olanzapine, quetiapine, risperidone
Stimulants/appetite suppressants	Amphetamines, phentermine, fenfluramine, and dexfenfluramine
Antimigraine drugs	Triptans, carbamazepine, ergot alkaloids, valproic acid
Miscellaneous	Dextromethorphan, cyclobenzaprine, tryptophan, lysergic acid diethylamide, chlorphenamine
Drug combinations	
MAOIs + SSRIs or SNRIs or TCAs or opiates	Imipramine + tranylcypromine Phenelzine + meperidine
SSRIs + MAOIs or TCAs or SNRIs or opiates or triptans	Fluoxetine + carbamazepine or phentermine or fentanyl
SNRIs + TCAs or MAOIs or opiates or triptans	Venlafaxine + lithium or calcineurin inhibitors or mirtazapine or tranylcypromine
Antibiotics/antifungals + SSRIs	Linezolid + SSRIs or tapentadol Fluconazole + citalopram Ciprofloxacin + methadone + venlafaxine

DNRI, dopamine-norepinephrine reuptake inhibitor; MAOI, monoamine oxidase inhibitor; SNRI, Serotonin-norepinephrine reuptake inhibitor; SS, serotonin syndrome; SSRI, selective serotonin reuptake inhibitors; TCA, tricyclic antidepressants.

Based on FDA’s concern with triptans, the FDA mandated in 2019 that US prescribing information for lasmiditan (a selective 5-hydroxytryptamine-1F agonist; ditan) include the potential risk for SS. Since then, events reported as SS were identified during continued clinical development and post-marketing surveillance of lasmiditan. These events are reviewed in later sections.

### Clinical features and classification

SS can be variable in presentation but typically includes a clinical triad of mental status change (e.g., confusion, delirium, and agitation), autonomic instability (e.g., sweating, mydriasis, diarrhea, hyperthermia, tachycardia, and hypertension), and neuromuscular hyperactivity (e.g., tremor, and hyperreflexia) (5, 10). Patients with mild serotonin toxicity are normothermic, have mild neuromuscular symptoms (e.g., hyperreflexia, inducible clonus) and insomnia, anxiety, nausea, diarrhea, hypertension, and tachycardia (5, 9). Mild cases resolve in 1–3 days after serotonergic drugs are discontinued and do not progress to SS (9).

Patients with moderate serotonin toxicity are hyperthermic and show signs including opsoclonus, myoclonus, tremor, agitation, mydriasis, flushing, and diaphoresis (5, 9). Severe toxicity is characterized by severe hyperthermia, multi-organ failure, electrocardiographic changes, confusion, and coma (9). Severe toxicity is almost always serious and clinically categorized as SS. Mild to moderate toxicity is usually caused by an overdose of a single drug, multiple drugs at low dose, or an increase in therapeutic doses, whereas severe toxicity, i.e., SS, generally occurs with concomitant administration of ≥2 serotonergic drugs (5, 9).

### Diagnosis

The lack of objective diagnostic biomarkers increases the uncertainties around SS. Thus, it is a diagnosis of exclusion that requires careful clinical observations and recognition of signs and symptoms (5, 9). To assist with the clinical recognition of SS, two sets of diagnostic criteria are used: the Sternbach diagnostic criteria (18) and the Hunter decision rule (19). According to Sternbach criteria (18), SS is present in patients with ≥3 of the following symptoms (mental status change, agitation, myoclonus, hyperreflexia, diaphoresis, shivering, tremor, diarrhea, incoordination, and fever) in a patient with a recent addition or increase in a known serotonergic agent, the absence of other possible etiologies (e.g., infection, substance abuse, withdrawal etc.) and with no recent addition or increase of a neuroleptic agent. Although simple in application, Sternbach’s criteria have been criticized for being low on both sensitivity and specificity. For example, the criteria also include confusion, restlessness, and ataxia. Theoretically, a patient with delirium resulting from anticholinergic medications would meet criteria; the inclusion of ataxia and incoordination does not make sense in that SS does not cause cerebellar features, and any patient who is agitated and confused may appear “ataxic.” However, the requirement for the serotonergic agent may mitigate the overlapping clinical picture with an atropinic toxicity.

**Figure 1:**
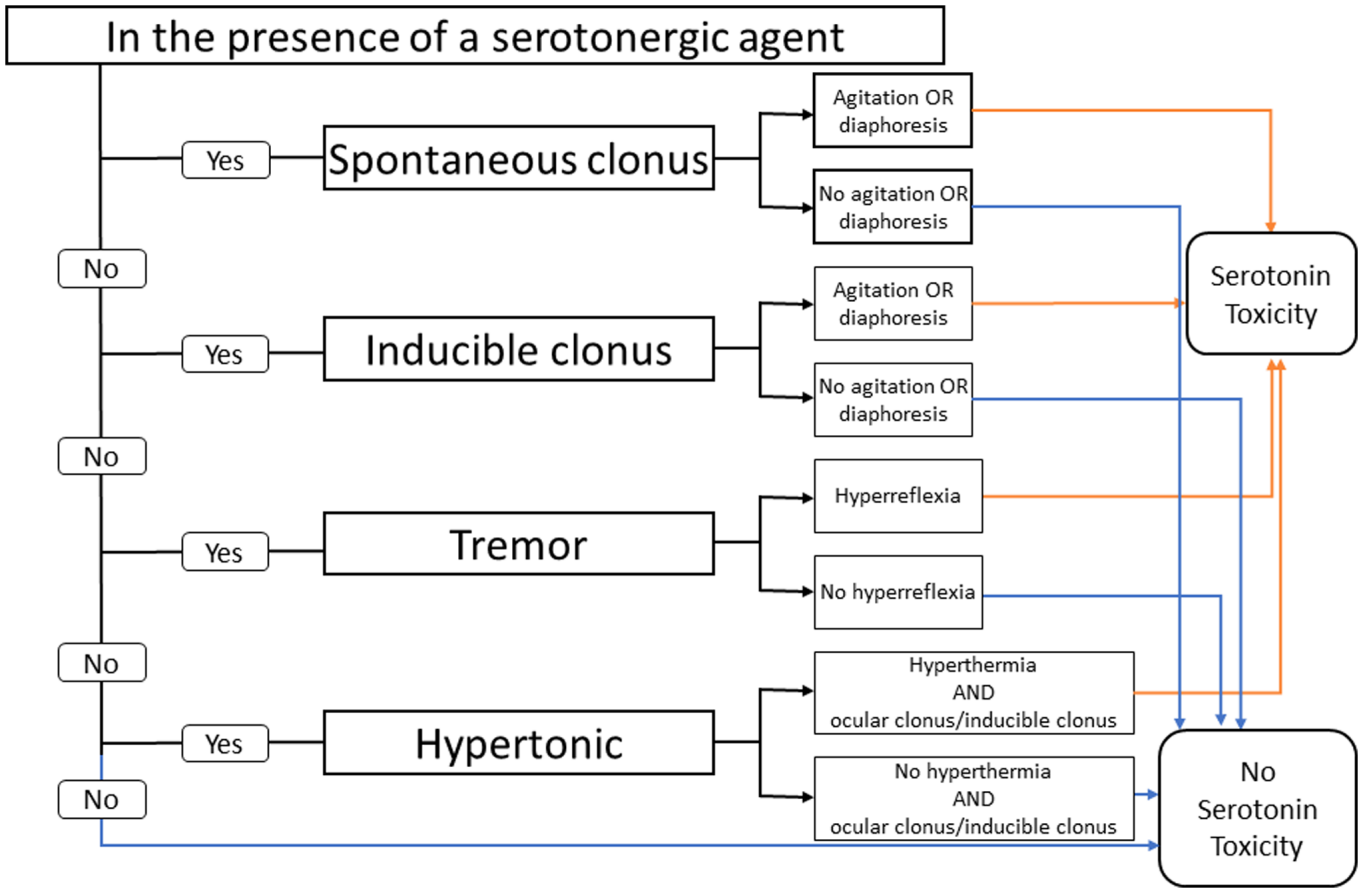
Hunter serotonin toxicity criteria: decision rules. Data from Buckley et al. (9).

In contrast, the Hunter criteria uses an algorithmic approach where the presence of clonus (inducible, spontaneous, and ocular) is the single most important diagnostic feature. The Hunter decision rules for diagnosing SS are found to be more sensitive (84% vs. 75%) and more specific (97% vs. 96%) than the Sternbach criteria (Figure 1) (19).

### Differential diagnosis

The list of SS differential diagnoses is long and includes conditions such as neuroleptic malignant syndrome, anticholinergic toxicity, malignant hyperthermia; thyrotoxicosis, and psychiatric conditions (e.g., anxiety disorder/panic attack, or catatonia).

### Management and prognosis

Prompt recognition of serotonin toxicity and syndrome is important, because early treatment is associated with a favorable prognosis. Discontinuation of serotonergic drugs can lead to resolution of symptoms in 24 to 72 h in mild cases. Initiation of supportive care, including administration of intravenous fluids and correction of vital signs, may be required in moderate-to-severe cases. Benzodiazepines are an essential component of treatment, particularly for managing agitation and tremor. Additionally, use of 5-HT_2A_ antagonists such as cyproheptadine or pizotifen may also be helpful for symptomatic relief. Aggressive management with intubation, sedation, and paralytics may be required in severe cases with muscle rigidity and hyperthermia.

### Experience with lasmiditan

Lasmiditan is a high-affinity and highly selective 5-hydroxytryptamine-1F agonist without significant action on the 5HT_1A_, 5HT_1B_, 5-HT_2A_, or 5HT_1D_ receptors and is approved for the acute treatment of migraine with or without aura. The pharmacological profile of lasmiditan shows that its agonist activity at the 5HT_1A_ and 5-HT_2A_ receptors is negligible within the range of clinical doses (20–23).

The most common adverse reactions reported with lasmiditan include dizziness, fatigue, paresthesia, and sedation (20, 24). Less common adverse reactions reported with lasmiditan include vertigo, incoordination, lethargy, visual impairment, feeling abnormal, palpitations, anxiety, tremor, restlessness, sleep abnormalities including sleep disturbance and abnormal dreams, muscle spasm, limb discomfort, cognitive changes, confusion, euphoric mood, chest discomfort, speech abnormalities, dyspnea, and hallucinations (20). Many of these adverse reactions are components of the SS, which can lead to confusion among providers and cause diagnostic errors. The following review discusses SS identified or reported with exposure to lasmiditan in clinical trials and following launch in the US.

## Methods

At the time of submission to the FDA (2018), an analysis of the lasmiditan clinical trial database was performed to understand the possible association of lasmiditan with SS. A detailed search for cases presenting with symptomatology potentially compatible with SS was conducted among five phase 2 and phase 3 completed clinical trials. Based on the pharmacology of lasmiditan and available clinical data, Lilly concluded that there was insufficient evidence to support an association between lasmiditan exposure and SS. The FDA, however, mandated a class labeling on the potential risk for SS in the REYVOW USPI (2019). In late 2019, a serious blinded case of SS requiring hospitalization was reported from an ongoing clinical trial. In 2020, after the completion of that trial and another study, Lilly re-reviewed the clinical trial data and available post-marketing reports on SS.

### Clinical trials

Eight lasmiditan trials including oral controlled and open labeled extension studies from phase 2 and phase 3 (*n* = 5,916) and the controlled intravenous (IV) trial (*n* = 88) were analyzed. In addition to cases reported as SS, potential cases were also identified based on a search of treatment emergent adverse events (TEAEs) within the Standardized Medical Dictionary for Regulatory Activities (MedDRA) Queries (SMQs) of neuroleptic malignant syndrome (SMQ 20000044; v21.0). The MedDRA preferred terms of “orthostatic hypotension,” “urinary incontinence,” “urinary retention,” “esophageal dysmotility,” “gastroparesis,” “diarrhea,” “fecal incontinence,” “constipation,” “muscle twitching,” “muscle stiffness,” and “muscle spasm” were also included in the search to ensure most clinical features of SS were captured.

Detailed medical review of identified cases by Lilly physicians included timing of events with respect to dosing with lasmiditan, nature, severity of symptoms, and use and timing of concomitant medications. Qualifying cases were further confirmed as SS using the predefined Sternbach and Hunter criteria (18, 19). In the current coding options within the standard medical dictionary (MedDRA), serotonin toxicity is not a preferred term and would default to SS in the adverse event surveillance system used by regulators and industry.

### Post marketing

As a part of routine surveillance and post-marketing commitment for the FDA, Lilly has been reviewing post-marketing reports on SS after exposure to lasmiditan.

The Lilly Safety System (LSS), which includes spontaneous reports, literature reports, regulatory authority reports, and reports from non-interventional studies, was searched for adverse events (AEs) using the MedDRA coding dictionary (v 23.0). For the current publication, reported cases of SS (out of an estimated 13,400 lasmiditan prescriptions) from the time of lasmiditan’s launch in the US on January 11, 2020 until a data cut-off on April 11, 2021 were included.

## Findings

### Clinical trials

Of the 6,004 patients treated with lasmiditan, 15 patients who reported at least one TEAE consistent with signs and symptom(s) possibly related to serotonin toxicity were identified. These cases were then reviewed in detail by three Lilly physicians, who determined that one case met both the Sternbach and Hunter criteria, and two cases potentially met the Sternbach criteria, while three cases were reported as SS with limited or no information to determine if they met either of the criteria.

Of the six cases, three were reported in the Phase 3 study H8H-MC-LAIJ (NCT03670810) (20). One case was reported as SS in the Phase 2 study COL-MIG-201/LAHM with IV lasmiditan (NCT00384774) (22), and one case each was identified based on reported symptomatology in the Phase 2 study COL-MIG-202/LAHO (NCT00883051) (25) and Phase 3 study COL-MIG-305/ LAHL (NCT02565186) (21, 23). All six cases were women, with an average age of 44 years. Four cases were non-serious, and two cases were serious, one of which required hospitalization with active treatment. All cases were reported recovered. Supplementary Table S2 provides the details of all the cases reported.

### Post marketing

Seventeen cases with symptom complexes that resembled SS were spontaneously reported during post-marketing surveillance for patients on treatment with lasmiditan.

Among the 17 cases, 12 occurred in women. Nine cases were reported as serious, one of which was reported by a consumer who had a previous history of SS. Of the eight cases that reported concomitant medications, six included specific serotonergic drugs (including cannabidiol oil and buspirone), and two mentioned unspecified antidepressant/SSRI intake. Ten of the 17 cases mentioned only ‘SS’ as an event without any additional description. Four cases had additional events reported that were known adverse reactions with lasmiditan. Three cases had adequate case descriptions for application of the predefined criteria. Of these three, two cases met the Sternbach and Hunter criteria, and one case met only the Sternbach criteria. Supplementary Table S3 provides details of post-marketing cases.

## Discussion

SS is the rare, serious, potentially life-threatening form of serotonin toxicity that is important to recognize and manage in a timely manner. The clinical presentation of serotonin toxicity can range from mild to severe. The Sternbach and Hunter criteria can be used in the formal diagnosis of SS. It is important for clinicians to be aware of both serotonin toxicity and SS and the application of the diagnostic criteria when required, as the clinical picture can be non-specific (5). Of these criteria, Hunter criteria are more sensitive and specific (12).

Various drug classes have been linked to serotonin toxicity and SS (Table 1). While the overall incidence of SS is unknown, SSRIs are the most commonly implicated drug class associated with this syndrome (11). Drugs with low affinity for 5-HT_1A_ and 5-HT_2A_ are thought to be unlikely candidates for precipitating serotonin toxicity because of their selectivity for other serotonin receptor subtypes and lack of influence on synaptic serotonin levels. However, many drugs with low affinity for 1A and 2A receptors have been implicated, which raises questions of whether we fully understand the pathophysiology of SS and the role serotonin agonists in its development (26). Case reports of SS have been reported for triptans and ditans. The biological plausibility remains elusive given the mechanism of action of the drugs and brings into question whether it is possible for any serotonin agonist to lead to SS *via* displacement of endogenous serotonin and resulting accessibility to 5-HT_1A_ or 5-HT_2A_ receptors.

While SS induced by lasmiditan is a rare event, given the putative pathophysiology of SS, the known pharmacological profile of lasmiditan, and the data from both clinical trial and post-marketing data, the acquired experience with the lasmiditan data review may help further characterize this syndrome.

Many of the known adverse events associated with lasmiditan can resemble SS. The presence of mild, expected serotonergic effects of the drug probably should not be diagnosed as SS if they do not meet Hunter/Sternbach criteria. Isolated or concurrent adverse events associated with serotonergic drugs are distinctly different from the symptom complex of serious SS, and, if suspected, should be reported as serotonin toxicity. Caution is imperative when there is dose titration or a combination of serotonergic agents co-prescribed, and clinicians need to be aware of the clinical syndrome to be able to suspect and diagnose it. The lack of diagnostic biomarkers for SS makes diagnosis highly dependent on clinicians’ observational skills. If serotonergic adverse events occur, it is important for a clinician to explore with further questioning to ascertain if the adverse events are isolated or accompanied by other symptoms indicative of possible serotonin toxicity and consider medication adjustment. However, overdiagnosis of SS in the absence of signs and symptoms meeting established criteria can adversely impact patients’ therapeutic approaches and understanding of the safety profile of a drug. Most cases reported or identified as ‘SS’ from lasmiditan clinical trial and post-marketing surveillance data were, in fact, mild to moderate, non-serious ‘serotonin toxicity,’ which were not recorded as such either due to a lack of awareness on the part of the reporter or limitations of the MedDRA coding dictionary that does not provide an option to code for toxicity in safety databases. Most post-marketing cases reported as ‘SS’ with lasmiditan lacked information on presenting symptomatology and recovered without any intervention, which may compromise diagnosis. Serotonergic drugs are generally safe, as evidenced by their widespread use in recent decades. Familiarity with the commonly anticipated and observed “serotonergic events” versus the rare syndromic manifestation will help prescribers from both under- or overreporting the syndrome.

From a research perspective, further studies are required to understand the association of serotonergic agonists used for the acute treatment of migraine and the pathophysiology of SS. Until then, it is imperative that we accurately characterize and record serotonin toxicity versus syndrome with these medications and to utilize established and valid diagnostic criteria.

## Conclusion

SS is a rare complication associated with lasmiditan administration. Awareness of the clinical features and knowledge of SS are key for accurate recognition, diagnosis, and appropriate management of patients. Prescribers should be aware of the medications associated with SS, the valid diagnostic criteria, the increased risk of serotonin toxicity when multiple serotonergic medications are used concomitantly, and the symptoms associated with the continuum from mild-to-severe serotonin toxicity and the rarer, serious SS to mitigate under- or over diagnosis. Additionally, patient education about the symptoms of SS can aid in early diagnosis and treatment, thereby preventing complications and progression to severe conditions.

## Data availability statement

The original contributions presented in the study are included in the article/Supplementary material, further inquiries can be directed to the corresponding author.

## Ethics statement

The studies involving humans were approved by an institutional review board or independent ethics committee at each of the study sites. The studies were conducted in accordance with the local legislation and institutional requirements. Written informed consent for participation was not required from the participants or the participants’ legal guardians/next of kin in accordance with the national legislation and institutional requirements.

## Author contributions

AB: Conceptualization, Formal analysis, Writing – review & editing. ST: Formal analysis, Writing – review & editing. RK: Conceptualization, Formal analysis, Writing – original draft, Writing – review & editing. ED: Conceptualization, Formal analysis, Writing – original draft, Writing – review & editing. MV: Formal analysis, Writing – original draft, Writing – review & editing. SM: Formal analysis, Writing – original draft, Writing – review & editing.

## Abbreviations

5-HT: 5-hydroxytryptamine
AE: Adverse event
CNS: Central nervous system
FDA: Food and Drug Administration
IV: Intravenous
LSS: Lilly Safety System
MAOI: Monoamine oxidase inhibitor
MedDRA: Medical Dictionary for Regulatory Activities
SERT: Serotonin reuptake transporter protein
SSRI: Selective serotonin reuptake inhibitor
SMQ: Standardized MedDRA query
SNRI: Serotonin-norepinephrine reuptake inhibitors
TEAE: Treatment-emergent adverse events
