# Supplementary material for: Serotonin syndrome in the acute treatment landscape of migraine: the lasmiditan experience
Source: Front Neurol. 2023 Oct 27;14:1291102. doi: 10.3389/fneur.2023.1291102 (PMC10641788; doi:10.3389/fneur.2023.1291102)
Supplement: Supplementary file 1 [file Data_Sheet_1.PDF]

Supplemental Tables and Figures.

**Supplemental Table 1** Serotonin receptors and their functions.

| Receptor subtype | Location | Function |
|---|---|---|
| 5-$HT_{1A}$ | Central Nervous System (CNS) | neuronal inhibition, behavioral effects |
| 5-$HT_{1B}$ | CNS | presynaptic inhibition, behavioral effects |
| | Vascular | pulmonary vasoconstriction |
| 5-$HT_{2A}$ | CNS | neuronal excitation, behavioral effects, and learning |
| | Smooth muscle | contraction, vasoconstriction/dilatation |
| | Platelets | aggregation |
| 5-$HT_{2B}$ | Stomach fundus | |
| 5-$HT_{2C}$ | CNS | choroid plexus, cerebrospinal fluid secretion |

| Receptor subtype | Location | Function |
|---|---|---|
| 5-$HT_3$ | Sensory and enteric nerves | |
| 5-$HT_4$ | CNS and myenteric neurons | gastrointestinal motility |
| 5-$HT_{5A}$ | CNS | |
| 5-$HT_6$ | CNS | |
| 5-$HT_7$ | CNS | |
| | Blood vessels | |
| | Gastrointestinal tract | |

Adapted from Mohammad-zadeh, et al 2008 (26).

**Supplemental Table 2:** Lasmiditan clinical trial cases with a treatment-emergent adverse event consistent with symptoms of serotonin syndrome

| Gender (Age, years) | Case summary (as reported) | Concomitant medications | Seriousness; causality |
|---|---|---|---|
| **Female (23)** | Patient with a medical history of mild depressive episode and moderate generalized anxiety disorder experienced moderate dizziness, moderate paranesthesias of both upper and lower legs and feet, and moderate agitation and akathisia 15 mins after receiving her first dose of study drug. Following which, she reported moderate myoclonus of both arms and legs, blurred vision, bilateral tinnitus, impaired coordination of both arms, sweating, unclear and slurred speech, and cervical dyskinesia. Symptoms lasted for approximately 21 hours. | Desogestrel, ibuprofen, metoclopramide and naratriptan | Serious with hospitalization and intervention required. Lorazepam (1mg) as corrective treatment for dizziness, paresthesia, agitation, and myoclonus was prescribed. The patient was sent to the emergency room. |

| Gender (Age, years) | Case summary (as reported) | Concomitant medications | Seriousness; causality |
|---|---|---|---|
| **Female (39)** | Patient with a history of hypothyroidism experienced tremors, nightmares, illusions, facial paresis, derealization, and agitation with moderate severity. Dizziness and ataxia were severe. All events began 25 minutes post the study drug and lasted from 1.5 hours to about 36 hours. | Concomitant serotonergic agents were not reported, except for agitation which was ongoing when frovatriptan was taken. | Not serious and no intervention required |
| **Female (61)** | Patient with medical history of depression, hypertension, and neck pain experienced muscle twitching, tremors, and agitation that started 79 minutes, 51 minutes, and 51 minutes post the study drug, respectively. The events lasted from 10 minutes to 1 hour. | Amoxicillin/clavulanic acid (BID) | Not serious and no intervention required |

| Gender (Age, years) | Case summary (as reported) | Concomitant medications | Seriousness; causality |
|---|---|---|---|
| **Female (52)** | Patient experienced moderate serotonin syndrome and moderate flushing 3 min after dosing. Dizziness was experienced 17 h after dosing. The symptoms lasted for 12 min to 10 hours. | Concomitant serotonergic agents were not reported. | Not serious and no intervention required |
| **Female (37)** | Patient with migraine experienced auditory hallucinations, agitation, and tachycardia, followed by mental confusion and finally muscular stiffness and not being able to walk post 2 hours of the study drug. Events began to decline after 3 hours, with only walking difficulty enduring. | Concomitant serotonergic agents were not reported. | Not serious and no intervention required |
| **Female (51)** | Patient with a medical history of migraine with aura, tension-type headaches, and fibromyalgia. Hallucinations of people, objects, and shapes, | Duloxetine and mirtazapine | Not serious and no intervention required |

| Gender (Age, years) | Case summary (as reported) | Concomitant medications | Seriousness; causality |
|---|---|---|---|
| | bilateral muscle twitching in the hands, confusion, and disorientation were experienced 25 minutes post the last dose. Symptoms lasted for approximately 2 hours. | | |
| **Female (35)** | Mild hot flushes were experienced by the patient, 36 minutes post the first dose of lasmiditan and lasted for 30 minutes. The patient experienced moderate tremor 1 hr and 6 min after the first dose of the study drug that lasted for 1 hr, and moderate somnolence (“drowsiness”), which began 2 hours and 6 minutes after dosing and lasted 2 hours. Medical history included increased blood cholesterol levels. | Sumatriptan, Paracetamol, Eugynon | Not serious; Concomitant serotonergic agent use was reported, but concurrent use with lasmiditan was unknown. |

| **Gender (Age, years)** | **Case summary (as reported)** | **Concomitant medications** | **Seriousness; causality** |
|---|---|---|---|
| **Female (60)** | Patient with a medical history of anxiety, ADHD, epistaxis, gastric ulcer, headache, hypothyroidism, hysterectomy, inguinal hernia, osteoarthritis, post menopause, and umbilical hernia experienced mild visual defect, mild flushing, moderate dyskinesia, moderate discomfort, and somnolence post the study drug. The symptoms lasted for 45 min to 2 hours. | Sumatriptan, progesterone, methylphenidate hydrochloride levothryoxine, estradiol, and Axotal (acetylsalicyslic acid) | Not serious; Concomitant serotonergic agent use was reported, but concurrent use with lasmiditan was unknown |
| **Female (44)** | Patient with a medical history of ankle arthroplasty, gastroesophageal reflux disease, hysterectomy, joint injury, knee arthroplasty, limb injury, obesity, plantar fasciitis, rotator cuff repair, and uterine leiomyoma experienced mild disturbance in attention, mild BP | Omeprazole | Not serious; Concomitant serotonergic agent use was reported |

| Gender (Age, years) | Case summary (as reported) | Concomitant medications | Seriousness; causality |
|---|---|---|---|
| | increased, moderate tremor, and severe paresthesias 40 minutes post study drug. The disturbance in attention and BP increased lasted 2.5 hours, and the tremor and paresthesia resolved the next day duration not specified). | | |
| **Female (46)** | Patient with a medical history of anemia, carpal tunnel decompression, carpal tunnel syndrome, cholecystectomy, and ligament sprain experienced dizziness, hyperhidrosis, paresthesia, somnolence, and tremors 30 minutes post study drug. | Concomitant serotonergic agents were not reported. | Not serious |
| **Male (54)** | Patient with a medical history of benign prostatic hyperplasia and seasonal allergies experienced mild | Zolmitriptan (rescue | Not serious |

| Gender (Age, years) | Case summary (as reported) | Concomitant medications | Seriousness; causality |
|---|---|---|---|
| | hypertonia 40 minutes post study drug and lasted for 20 minutes. | medication 24 hours after dose)<br><br>acetylsalicylic acid<br><br>(rescue medication 7 hours and 10 min after dose) | |
| **Female (46)** | Patient experienced flushing, dizziness, nausea, balance disorder, and diarrhea which began 27 minutes, 28 minutes, 29 minutes, 39 minutes, and 39 minutes post study drug, respectively. The symptoms lasted for 17 minutes to 1 hour and 5 minutes. | Concomitant serotonergic agents were not reported. | Not serious |

| Gender (Age, years) | Case summary (as reported) | Concomitant medications | Seriousness; causality |
|---|---|---|---|
| **Female (33)** | Patient experienced mild tremors and mild visual hallucinations 25 minutes post the study drug that lasted for 5 hours. Mild diarrhea began the following day. | Concomitant serotonergic agents were not reported. | Not serious |
| **Female (23)** | Patient experienced mild blurred vision, tremor, and blurred vision on the last day of the study drug. | Ongoing cyclobenzaprine (muscle relaxant) | Not serious |
| **Female (40)** | Patient experienced somnolence immediately after receiving study drug. Mild chills were observed the following day accompanied by mild stiffness of the neck. | Concomitant serotonergic agents were not reported. | Not serious |

Abbreviations: ADHD = attention-deficit/hyperactivity disorder; BID = twice daily; BP = blood pressure; TEAE = treatment-emergent adverse events.

Supplemental **Table 3:** Summary of lasmiditan post-marketing cases reported as serotonin syndrome

| **Gender (Age, years)** | **Case summary** | **Concomitant medications** | **Seriousness; causality** |
|---|---|---|---|
| **The events observed that met the Sternbach and the Hunter criteria** | | | |
| **Female (30)** | Patient with a history of chronic migraine, bipolar disorder, attention deficit hyperactivity disorder experienced serotonin syndrome-like symptoms 30 min post lasmiditan dose. The symptoms lasted for 4 to 5 hours. Symptoms included altered level of consciousness, autonomic dysfunction, myoclonus, diarrhea, nausea, confusion, and shaking. | Zolmitriptan,(tablet and nasal spray), escitalopram, botulinum toxin type A, lamotrigine, levothyroxine, lisdexamfetamine, topiramate, naproxen, spironolactone, zolpidem, withania somnifera root, ubrogepant, adapalene topical, amphetamine dextroamphetamine, bupropion, naltrexone and magnesium oxide. | Serious, but no intervention provided |
| **Female (39)** | Patient with a history of major depressive disorder, anxiety, idiopathic chronic constipation, and gastroparesis experienced | Alprazolam, lubiprostone, aspirin, bupropion hydrochloride, buspirone hydrochloride, methocarbamol, clarithromycin, omeprazole, | Serious. Diphenhydramine 25 mg, promethazine 12.5 |

| | | | |
|---|---|---|---|
| | dizziness and lethargy within 1 hour of lasmiditan treatment. The symptoms progressed to nausea, fever (temperature of 100.3 F), muscle twitches, vomiting, loss of focus and concentration, light sensitivity, jaw tightness, bad diarrhea, racing thoughts, loss of speech, loss of movement, stumbling, inadequate coordination, and worsened agitation. Following treatment, patient was in a state of hysteria, and reported memory problems the following day. Patient also reported hyperthermia, altered level of consciousness, myoclonus, and vertigo (all non-serious) which lasted for days. | spironolactone, fremanezumab, and botulinum toxin type A. | mg, and lorazepam 0.5 mg was provided. |

**The events observed that met the Sternbach criteria**

| | | | |
|---|---|---|---|
| **Female (30)** | Patient with a history of serotonin syndrome on selective serotonin reuptake inhibitors (SSRI) experienced several symptoms including “tripping out,” unable to read, could not form sentences, could not understand what was happening, unable to walk straight, violent tremors and body shaking, sudden jerky movements with her jaw shaking while trying to speak, unable to control her jaw while talking, had a racing heart and sensation of choking, unable to remember words and vomiting, feeling like she was falling when laying down, dizziness and feeling the room spinning, agitated mind, unable to process what she would see on TV or cell phone, hallucination patterns in the dark, and | Not provided | Serious, but no intervention required |

| | | | |
|---|---|---|---|
| | seeing a purple or green light trail when looking at lights. The symptoms lasted for 2 hours. | | |
| **The events observed that did not meet the Sternbach and the Hunter criteria** | | | |
| **Female** | Patient experienced increased heart rate and muscle tightness up for to 6 hours at night. | Cannabidiol (CBD) oil 20 mg nightly. Other concomitant medications are not provided. | Serious, possibly due to drug interaction |
| **Female (36)** | Patient experienced feeling terrible and very dizzy, with no spatial awareness, lack of coordination, and difficulty speaking. The symptoms lasted for 10 hours. | Doxepin 75 mg each evening | Serious, possibly due to drug interaction |
| **Female (45)** | Patient experienced homicidal hallucinations post treatment with lasmiditan. | Multiple unspecified medications including an antidepressant | Serious, but no intervention provided |
| **Female** | Patient who was a frequent visitor of the emergency room (ER) went to the ER after the | Unspecified SSRIs | Serious, but no intervention provided |

| | | | |
|---|---|---|---|
| | first dose of lasmiditan for serotonin syndrome-like symptoms. | | |
| **Unknown** | Patient with a history of gastroparesis experienced serotonin toxicity after taking lasmiditan for the first time. Only limited case information was available. | Not reported | Serious, but no intervention provided |
| **Unknown** | Patient experienced serotonin syndrome post treatment with lasmiditan. Only limited case information was available. | Not reported | Serious, but no intervention provided |
| **Female** | Limited information provided but serotonin syndrome-like symptoms appeared after lasmiditan treatment. After the physician discontinued the patient' serotonin medication, symptoms stopped. Unknown if the patient continued to take lasmiditan. | Unspecified serotonin medication | Non-serious |

| | | | |
|---|---|---|---|
| **Female** | Patient experienced sweating after some time of lasmiditan treatment. | Not reported | Non-serious |
| **Female (25)** | Patient experienced nausea and vomiting after taking lasmiditan. The physician reported it, as the patient was not receiving any other concomitant medication. | Not reported | Non-serious |
| **Female** | Patient experienced serotonin syndrome-like symptoms, but limited information was available on time of onset and exact symptoms. | Not provided | Non-serious |
| **Female (38)** | Patient with a history of rheumatoid arthritis was brought to the ER and diagnosed with serotonin syndrome after her first dose of lasmiditan. Lasmiditan intake was discontinued. | Galcanezumab and other unspecified products | Non-serious |

| | | | |
|---|---|---|---|
| **Male (82)** | Patient experienced anxiety and visual disturbances after his first dose of lasmiditan. Lasmiditan intake was discontinued, and the patient was advised to go to the ER. The physician was unsure if the symptoms signified serotonin syndrome. | Not provided | Non-serious |
| **Unknown** | Patient experienced serotonin syndrome-like symptoms after first dose of lasmiditan, but there was limited information on the available time of onset and exact symptoms. | Sertraline hydrochloride | Non-serious; possibly due to drug interaction |
| **Unknown** | Patient experienced serotonin syndrome-like symptoms. | Not reported | Non-serious |

Abbreviations: TEAE = treatment-emergent adverse event.
